# Design and Evaluation of a Custom-Made Electromyographic Biofeedback System for Facial Rehabilitation

**DOI:** 10.3389/fnins.2022.666173

**Published:** 2022-03-04

**Authors:** Kathrin Machetanz, Florian Grimm, Ruth Schäfer, Leonidas Trakolis, Helene Hurth, Patrick Haas, Alireza Gharabaghi, Marcos Tatagiba, Georgios Naros

**Affiliations:** ^1^Department of Neurosurgery and Neurotechnology, Eberhard Karls University of Tübingen, Tübingen, Germany; ^2^Institute for Neuromodulation and Neurotechnology, Eberhard Karls University of Tübingen, Tübingen, Germany; ^3^Department of Hand, Plastic, Reconstructive and Burn Surgery, BG Clinic, Tübingen, Germany

**Keywords:** facial nerve palsy, electromyography, biofeedback, physical computing platform, vestibular schwannoma

## Abstract

**Background:**

In the rehabilitation of postoperative facial palsy, physical therapy is of paramount importance. However, in the early rehabilitation phase, voluntary movements are often limited, and thus, the motivation of patients is impacted. In these situations, biofeedback of facial electromyographic (EMG) signals enables the visual representation of the rehabilitation progress, even without apparent facial movements. In the present study, we designed and evaluated a custom-made EMG biofeedback system enabling cost-effective facial rehabilitation.

**Methods:**

This prospective study describes a custom-made EMG system, consisting of a microcontroller board and muscle sensors, which was used to record the EMG of *frontal* and *zygomatic* facial muscles during *frowning* and *smiling*. First, the mean EMG amplitudes and movement onset detection rates (ACC) achieved with the custom-made EMG system were compared with a commercial EMG device in 12 healthy subjects. Subsequently, the custom-made device was applied to 12 patients with and without postoperative facial paresis after neurosurgical intervention. Here, the ratio [laterality index (LI)] between the mean EMG amplitude of the healthy and affected side was calculated and related to the facial function as measured by the House and Brackmann scale (H&B) ranging from 1 (normal) to 6 (total paralysis).

**Results:**

In healthy subjects, a good correlation was measured between the mean EMG amplitudes of the custom-made and commercial EMG device for both *frontal* (*r* = 0.84, *p* = 0.001) and *zygomatic* muscles (*r* = 0.8, *p* = 0.002). In patients, the LI of the frontal and zygomatic muscles correlated significantly with the H&B (*r* = −0.83, *p* = 0.001 and *r* = −0.65, *p* = 0.023). The ACC of the custom-made EMG system varied between 65 and 79% depending on the recorded muscle and cohort.

**Conclusion:**

The present study demonstrates a good application potential of our custom-made EMG biofeedback device to detect facial EMG activity in healthy subjects as well as patients with facial palsies. There is a correlation between the electrophysiological measurements and the clinical outcome. Such a device might enable cost-efficient home-based facial EMG biofeedback. However, movement detection accuracy should be improved in future studies to reach ranges of commercial devices.

## Introduction

Neurorehabilitation of peripheral facial palsy (e.g., after neurosurgical procedures) is challenging, and the efficiency of the hitherto applied rehabilitation techniques is still under debate (Shafshak, 2006; Pereira et al., 2011; Teixeira et al., 2011; Baricich et al., 2012). Until now, the most common approach to facial neurorehabilitation is repetitive physical therapy (Pereira et al., 2011; Teixeira et al., 2011; Van Landingham et al., 2018). In severely affected patients, however, motivation to participate in long-lasting rehabilitation programs is impeded by the lack of apparent voluntary movements or the lagged clinical improvement. Continuous feedback of electromyographic (EMG) muscle activity enables visual representation of the rehabilitation progress, even without apparent gross facial movements. Such biofeedback-based neurorehabilitation has been shown to maintain the motivation of the patients in the context of stroke rehabilitation (Naros et al., 2016; Tamburella et al., 2019). In line, the use of EMG biofeedback has been suggested for facial rehabilitation several years ago (Booker et al., 1969; Brown et al., 1978; Balliet et al., 1982; Ross et al., 1991; Cronin and Steenerson, 2003). However, studies on the therapeutic benefit of EMG feedback are still controversial (Ross et al., 1991; Nakamura et al., 2003; Dalla-Toffola et al., 2005; Pourmomeny et al., 2014). A common flaw of these studies was a limitation of the training period to a few weeks or a low frequency of training sessions per week [e.g., Ross et al. (1991) two sessions per week for 2 weeks, one session per week for 6 weeks, and two sessions per month for 10 months for a total treatment duration of 1 year]. Furthermore, the validity of previous studies on EMG biofeedback training is limited by small patient cohorts (<15 patients) (Brown et al., 1978; Balliet et al., 1982; Ross et al., 1991; Volk et al., 2014). This can be partially attributed to the high acquisition costs and the low availability of EMG devices necessitating the biofeedback training to be offered mainly in therapy centers (Ross et al., 1991; Dalla-Toffola et al., 2005). New technological solutions are demanded for mobile EMG systems keeping size, costs, and power consumption reasonable and enabling home-based biofeedback therapy.

The development of single-board microcontroller and systems-on-a-chip solutions is the basis of current open-source physical computing (PhysComp) platforms, such as the Arduino^®^ or Arduino-like systems. Initially created to teach students electronics and subsequently widespread in the so-called do-it-yourself community, there is an increasing interest of the medical sector in microcontroller boards facilitating the development of cost-effective EMG devices. First studies have demonstrated comparable results between custom-made PhysComp and commercial EMG devices (Supuk et al., 2014; Heywood et al., 2018; del Toro et al., 2019; Prakash et al., 2019; Toro et al., 2019). However, until now, studies have mostly examined large muscle groups such as the vastus lateralis muscle or the rectus femoris muscle. The applicability of these systems to small muscle groups, such as the facial musculature, has not yet been clarified. In this respect, the present study aimed to evaluate a cost-effective, custom-made PhysComp EMG device for facial rehabilitation in healthy subjects as well as in patients with postoperative facial palsies.

## Materials and Methods

### Study Cohort

This prospective, proof-of-concept study included 12 healthy subjects (29.9 ± 7.0 years, 7 women) to evaluate a custom-made EMG system (Arduino^®^ Uno WiFi Rev 2.0, MA, United States + MyoWare™ Muscle Sensor, Advancer Technologies, NC, United States) in comparison with a commercial EMG amplifier (BrainAmp ExG, Brain Products GmbH, Gilching, Germany). In a second step, 12 patients (55.1 ± 9.0 years, 5 women), with and without postoperative facial palsies, were included within the first 6 days after neurosurgical removal of a unilateral vestibular schwannoma (right-sided: *n* = 6, left-sided: *n* = 6) to examine the correlation between the degree of facial palsy and facial EMG activity as well as to prove the feasibility of an EMG biofeedback with the custom-made system. Tumor size and extension were classified according to the Hannover classification (T1: purely intrameatal, T2: intra- and extrameatal, T3: filling the cerebellopontine cistern, and T4: compressing the brainstem) because previous studies demonstrated a significant correlation between tumor size and the grade of postoperative facial palsy (Sughrue et al., 2010; Falcioni et al., 2011; Breun et al., 2019). Cohorts’ characteristics are shown in Table 1. The study was approved by the local ethics committee of the Eberhard Karls University of Tübingen and performed in accordance to the Declaration of Helsinki. All participants gave a written informed consent.

**TABLE 1:** Cohort characteristics.

	Healthy subjects	VS patients
	
	*n* = 12	*n* = 12
**Age**	29.91 ± 6.69	55.08 ± 9.02
**Gender (f:m)**	7:5	5:7
**Tumor size** *T1* *T2* *T3* *T4*		0/12 (0.0%) 5/12 (41.7%) 3/12 (25.0%) 4/12 (33,3%)
**Side of facial paresis** *Left* *Right*		6/12 (50%) 6/12 (50%)
**Facial function**		
*H&B*		
*I*	12/12 (100%)	4/12 (33.3%)
*II*	–	2/12 (16.7%)
*III*	–	3/12 (25.0%)
*IV*	–	1/12 (8.3%)
*V*	–	2/12 (16.7%)
*VI*	–	–

*VS, vestibular schwannoma; H&B, House and Brackmann scale.*

### Electromyographic Sensor Systems

**FIGURE 1:**
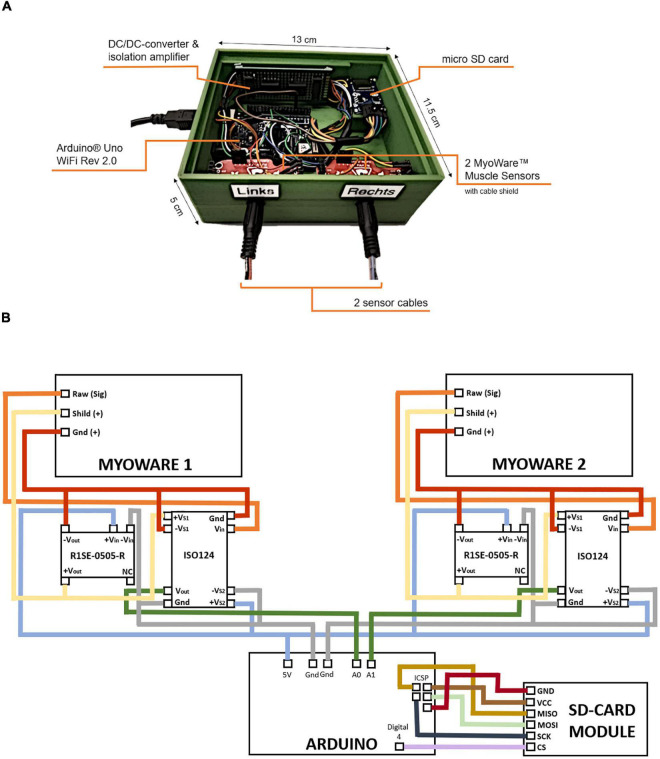
Custom-made electromyographic (EMG) device. Components and dimensions (A). The setup integrates an Arduino^®^ Uno WiFi Rev 2.0, two sensor chips (MyoWare™ Muscle Sensor, Advancer Technologies, NC, United States), two DC/DC converter (R1SE-0505-R, Recom Power GmbH, Gmunden, Austria), and isolation amplifiers (ISO124, Texas Instruments Incorporated, Dallas, TX, United States) as well as one SD card module (B).

The custom-made EMG system (Figures 1A,B) combined two sensor chips (MyoWare™ Muscle Sensor, Advancer Technologies, NC, United States) with a physical computing platform (Arduino^®^ Uno WiFi Rev 2.0, MA, United States). The MyoWare muscle sensor (4 × 52.3 × 20.7 mm) is a system-on-a-chip (SoC), whose embedded electrodes allow a direct attachment to the muscle. The amplifier is specified by an input impedance off 100 GΩ, a common mode rejection rate (CMRR) of 110 dB, and a supply voltage (V_*in*_) of 2.9–5.7 V. The amplifier gain is adjustable *via* a potentiometer (V_*out*_/V_*in*_ = 201 * R_*gain*_/1 kΩ; R_*gain*_ ranges between 0.01 Ω and 100 kΩ). In the presented EMG system, data processing and analysis were warranted by using the raw output mode, which does not enable an adaptation of the output voltage by means of a potentiometer (i.e., output is centered about an offset voltage of + V_*in*_/2 and ranges between 0 and + V_*in*_). For better subject and patient comfort, muscle sensors were supplemented by cable shields (MyoWare cable shields, Advancer Technologies, NC, United States) and sensor cables (Advancer Technologies, NC, United States). Connection to the Arduino WiFi Rev 2.0 was made *via* a DC/DC converter and an isolation amplifier (R1SE-0505-R, Recom Power GmbH, Gmunden, Austria and ISO124, Texas Instruments Incorporated, Dallas, TX, United States). The Arduino WiFi Rev 2.0 is a physical computing platform incorporating open-source hard- and software. It integrates an ATmega4809 8-bit microcontroller, a self-contained SoC WiFi-module, and 14 digital in- and outputs as well as 6 analog input pins. The ATmega4809 8-bit microcontroller integrates a 16-channel, high-speed 10-bit analog-to-digital converter (ADC), which renders the input voltages between 0 and the operating voltage (i.e., 5 V) into integer values between 0 and 1023. These integers were processed by custom-written Arduino- and MATLAB-based algorithms for a basic online feedback. The integer data were transferred *via* the serial port using the MATLAB functions fopen() and fscanf(). Furthermore, data storage of the input sensor data was ensured by transferring the data from the Arduino^®^ to a micro-SD card and a wire-connected computer (ThinkPad T495, Lenovo Group Limited, Beijing, China) with a sampling frequency of 100 Hz. The case of the EMG device (50 × 115 × 130 mm) was constructed in a computer-aided design (CAD) program (NX 10, Siemens, Munich, Germany) and produced using an in-house 3D-printer (Prusa i3 MK3S, Prusa Research a.s., Prag, Czech Republic) and polylactide filament. Overall, the described custom-based EMG device costs about $150 in total.

The commercial Brain Products system (Brain Products GmbH, Gilching, Germany) processes the EMG data through a 16-channel BrainAmp ExG amplifier (68 × 160 × 187 mm), which is powered with a rechargeable battery and can record signals with a sampling rate up to 5,000 Hz and a DC offset tolerance of ± 300 mV. The amplifier is specified by an input impedance off 10 MΩ and a common mode CMRR of ≥ 100 dB. The ADC has a bit width of 16 bit. The input data are transmitted from the electrodes *via* an ExG AUX box and duplex fiber optic cables to a computer where the data are processed, visualized, and stored using the BrainVision software. These commercial soft- and hardware packages cost up to several thousand dollars.

### Experimental Procedure in Healthy Subjects

**FIGURE 2:**
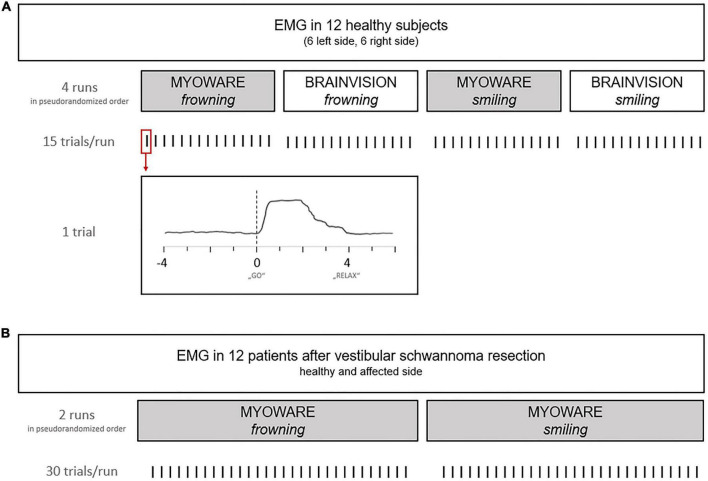
Overview of the experimental procedure during EMG recordings. During the first exploratory step (A) in healthy patients, muscle activity was recorded by a commercial and the custom-made device. The second part of the study (B) investigated facial EMG in patients after vestibular schwannoma (VS) resection.

In a parallel-group design, 12 healthy subjects were pseudorandomized in two groups [i.e., EMG of the left (*n* = 6) or right (*n* = 6) side of the face] and participated in one session to evaluate the custom-designed EMG system. Each session lasted approximately 15–20 min consisting of four runs of 15 trials/movements (Figure 2A). Each trial started with a 4-s *REST* period. After a visual command (“GO”), subjects were asked to perform either *frowning* or *smiling* for a period of 4-s (*MOVE*). Finally, the *MOVE* period was finished by the visual command (“RELAX”). EMG activity of the frontal and zygomatic muscles was measured during the movements *frowning* and *smiling* once by the low-cost EMG system (100-Hz sampling rate) and once by the Brain Products system (1,000-Hz sampling rate). The different movement sequences/runs (Figure 2A) were performed in a pseudorandomized order. The same surface electrodes (Kendall, Covidien, Ireland) were used for both sensor systems. In this first part of the study, healthy subjects received no feedback on their performance. Movement onset detection rate (i.e., accuracy in %, ACC) was analyzed offline as described in a separate passage for both healthy subjects and patients.

### Experimental Procedure in Patients

In 12 patients, facial EMG activity and the degree of facial nerve palsy were assessed according to the House and Brackmann scale (H&B) within the first 6 days after resection of a vestibular schwannoma (VS). The H&B score classifies overall facial function into ranges from 1 (normal) to 6 (total paralysis) based on the assessment of, e.g., eye closure, forehead, and mouth movement (House and Brackmann, 1985). The scale does not include regional subdomain scores. However, due to its simplicity, it is the most commonly used scale for assessing peripheral facial paresis. The EMG activity of the frontal and zygomatic muscles of the patients was derived *via* surface electrodes (Kendall, Covidien, Ireland) simultaneously on both sides of the face, while patients had to perform the movements *frowning* and *smiling* (i.e., two runs) in a pseudorandomized sequence. Each movement was performed 30 times (i.e., trials) in a session lasting approximately 15–20 min (Figure 2B). Each trial started with a 4-s *REST* period. After a visual command (“GO”), the patients were asked to either *frown* or *smile* for a period of 4-s (*MOVE*). Finally, the *MOVE* period was finished by the visual command (“RELAX”). Surface EMG of the healthy and the affected side was measured with the custom-made EMG system (100-Hz sampling rate) and automatically stored on a micro-SD card.

### Online Feedback Algorithm

**FIGURE 3:**
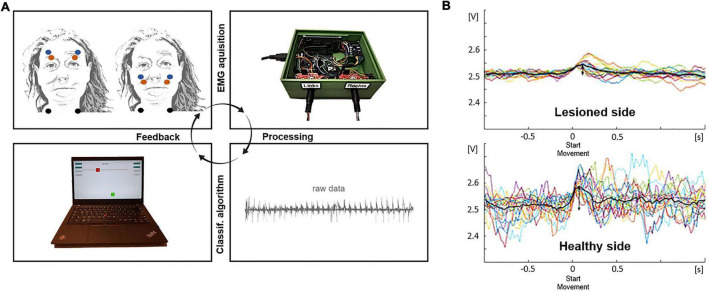
Feedback loop during facial rehabilitation and exemplary data. (A) Electrodes (red and blue: detecting electrodes, black: reference electrode) are positioned at the zygomatic and frontal muscles of the healthy and lesioned side (upper left). After recording the muscle activity with the sensor chip (upper right) and processing information with the microcontroller and different analysis algorithms in the Arduino IDE and MATLAB (lower right), information is transformed in a visual feedback for the patient (lower left). (B) Exemplary data shows individual and averaged trials of the lesioned and healthy side of the face.

In this second part of the study, the patients received visual feedback about their muscle activity *via* a computer screen after each trial of a run (i.e., 30 trials per run; two runs per patient: *frowning* and *smiling*) using a custom-written Arduino- and MATLAB-based algorithm (Arduino IDE, United States, 1.8.10 and Mathworks Ltd., United States, R2018b). In detail, the EMG signal of both halves of the face was feedbacked by two colored squares (green: left and red: right) on a computer screen. In case of significant movement-related EMG activity during *MOVE*, the position of the squares shifted to the top of the screen and provided visual feedback for 2 s before moving back to the initial position (Figure 3A). If no significant movement was detected during *MOVE*, squares were positioned at the bottom of the screen. To define the significance of movement, a basic online classification algorithm was applied to compare EMG amplitudes in the *REST* and *MOVE* period of each trial at this stage of the development. For online classification, the EMG signal was rectified, and the mean EMG amplitudes were acquired using a circular buffer of 400-ms length and a moving window of 200 ms. Depending on the current feedback period (*REST* or *MOVE*), these amplitude values were assigned to one of two data buffers. The *REST* buffer contained data of the past three *REST* periods, while the *MOVE* buffer contained data of the current *MOVE* period. At the end of each *MOVE* period, EMG contraction levels were classified between *MOVE* and *REST* using a non-parametric Kruskal–Wallis test between the data buffers. A *p*-value < 0.05 was considered as significant movement-related EMG activity resulting in corresponding visual feedback. Movement onset detection rate (i.e., accuracy in %, ACC) was analyzed offline as described separately for both healthy subjects and patients.

### Offline Analysis of Electromyographic Data

Offline data analysis was performed using custom-written scripts in MATLAB (Mathworks Ltd., United States, R2018b). Raw electromyographic data of both EMG systems were imported into MATLAB, rectified, and filtered using a low-pass filter with frequencies of 49 and 449 Hz for the custom-designed EMG device (100-Hz sampling rate) and the commercial EMG amplifier (1,000-Hz sampling rate), respectively. Subsequently, data were smoothed by applying a centered moving average filter with a moving average length of 10 samples. After selection of the time period to be analyzed (i.e., 15 movements in healthy subjects and 30 movements in patients) and visual specification of a movement-defining amplitude threshold, maximum peaks were automatically detected by the MATLAB function findpeaks(). Data were segmented into epochs from −0.5 to 1 s relative to the maximum EMG peak. Visual artifact correction was done, and selected epochs/trials were averaged, while epochs with artifacts were excluded from further analysis. Finally, the mean amplitudes of the *REST* and *MOVE* periods were subtracted (i.e., meanMOVE − meanREST) resulting in a movement-related EMG amplitude, referred to—dependent on the acquisition device—as MYO_*diff*_ (MyoWare) or BRAINV_*diff*_ (BrainAmp). A correlation analysis between absolute MYO_*diff*_ and BRAINV_*diff*_ values was performed in the first part of the study to compare EMG devices. In VS patients, after repeating the EMG analysis presented for both types of movement and both sides of the face (Figure 3B), the difference between the MYO_*diff*_ value of the healthy and affected side was calculated resulting in two values describing the laterality index (LI) for *frowning* and *smiling*. An LI > 1 corresponded to a higher mean amplitude on the affected compared with the healthy side, while an LI < 1 is correlated to a higher amplitude on the healthy side.

### Classification Accuracy of the Movement Onset

Movement onset detection rate (i.e., accuracy in %, ACC) was determined indicating the percentage of all trials (i.e., 15 in healthy subjects, 30 in VS patients) with a significant movement-related EMG activity (i.e., *p* < 0.05 in the Kruskal–Wallis test). ACC was analyzed offline and compared for each movement type (i.e., *frowning* and *smiling*), custom-based and commercial EMG device, as well as for the affected and healthy side in patients in the same way.

### Statistics

All statistical tests were performed using MATLAB (MathWorks Inc., Natick, MA, United States, R2018b) and SPSS (IBM SPSS Statistics for Windows, Version 26.0. Armonk, NY, United States IBM Corp.). Correlation analysis between Myoware amplitude (MYO_*diff*_; meanMOVE − meanREST in Myoware data) and BrainVision Amplitude (BRAINV_*diff*_; meanMOVE − meanREST in Brain Products data) in healthy subjects was based on two-tailed Pearson’s correlation coefficients, whereas the correlation between LI and H&B in patients was based on two-tailed Spearman’s correlation coefficient. Comparison of movement onset detection rates was performed using Student’s test. Finally, correlation analysis between ACC and H&B in VS patients was based on Kruskal–Wallis test. Data are shown as the mean ± standard deviation (SD). Values of *p* < 0.05 were considered significant.

## Results

The present study includes 12 healthy as well as 12 patients with one-sided vestibular schwannomas. The tumor extent according to the Hannover classification as well as the grade of facial palsy are illustrated in Table 1. Movement-related EMG activity of frontal and zygomatic muscles was detectable in all participants and with both EMG systems. A total of 4.4% (MyoWare) and 3.6% (BrainAmp) of the trials were excluded in healthy subjects, while 2.92 and 1.5% of the trials of the affected and healthy side of the face were excluded in patients. There were no side effects or undesired events during measurements and biofeedback.

### Comparison of Electromyographic Activity Measured by the Custom-Made and Commercial Device

**FIGURE 4:**
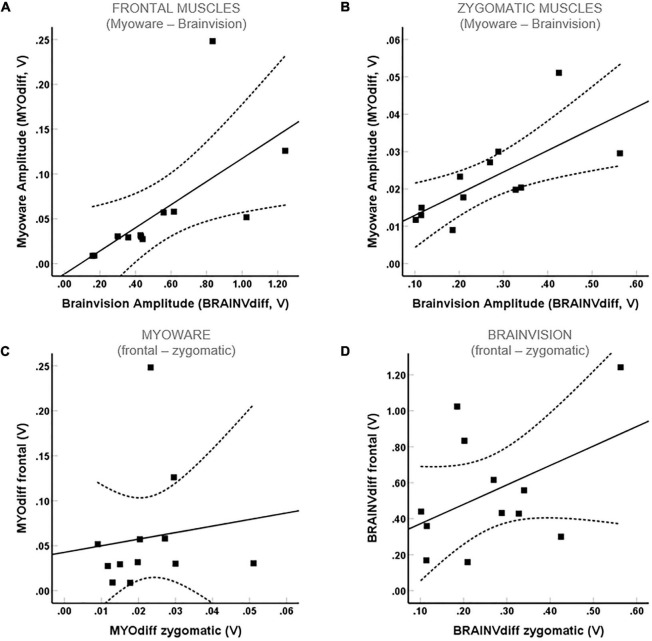
Correlation between EMG mean amplitudes measured by the commercial and custom-made EMG device in healthy patients. Significant correlations for frontal (A) and zygomatic (B) muscles were found, while there is no significant correlation between frowning and smiling either for the MYO_*diff*_ (C) or for the BRAINV_*diff*_ (D) mean amplitude.

Electromyographic activity of the frontal and zygomatic muscles was derived in a pseudorandomized order during the movements *frowning* and *smiling* using a custom-made and a commercial EMG system. There was a significant correlation between the MyoWare (MYO_*diff*_) and BrainAmp mean amplitude (BRAINV_*diff*_) for both *frowning* (*r* = 0.84, *p* = 0.001; Pearson’s correlation; Figure 4A) and *smiling* (*r* = 0.8, *p* = 0.002; Pearson’s correlation; Figure 4B) indicating a good consistency of EMG signal detection across devices. Furthermore, we were able to demonstrate good movement specificity for both devices as there was no significant correlation between *frowning* and *smiling* neither for the MYO_*diff*_ (*r* = 0.12, *p* = 0.703; Pearson’s correlation) nor for the BRAINV_*diff*_ mean amplitude (*r* = 0.45, *p* = 0.145; Pearson’s correlation; Figures 4C,D).

### Correlation Between the Electromyographic Activity and Facial Palsy

**FIGURE 5:**
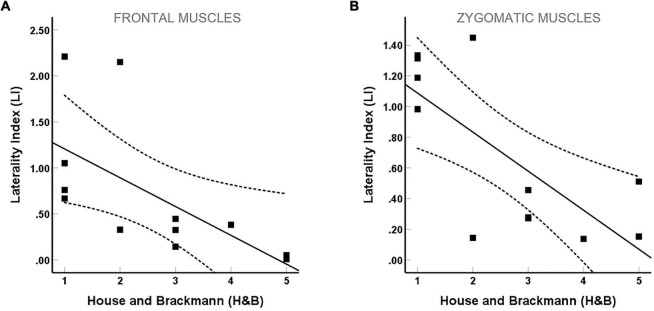
Correlation between the laterality index (LI) and facial palsy as measured by the House and Brackmann (H&B) scale. (A) Frontal and (B) zygomatic.

In 8 out of 12 patients (66.7%), postoperative facial nerve palsy with an H&B score II–VI occurred due to the VS resection (Table 1). The degree of facial nerve palsy correlated positively with tumor extent according to the Hannover classification (*H&B*: *r* = 0.68, *p* = 0.014; Spearman’s correlation). For both movements, a significant negative correlation between H&B and LI (*frowning*: *r* = –0.83, *p* = 0.001; *smiling*: *r* = –0.65, *p* = 0.023; Spearman’s correlation; Figures 5A,B) was detected, indicating a good congruence between the facial motor status and the EMG signals recorded by the custom-made EMG system. Furthermore, the LI of *smiling* and *frowning* correlated significantly (*r* = 0.69, *p* = 0.014; Spearman’s correlation).

### Detection of Movement Onset by the Biofeedback System

**FIGURE 6:**
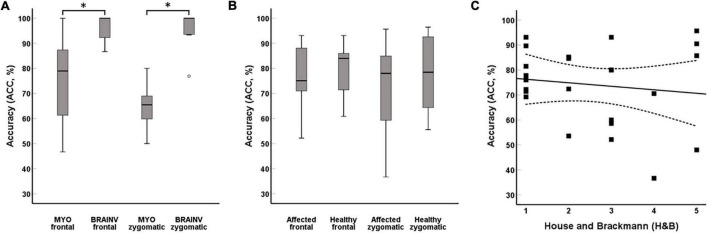
Classification incidence of movement onset. The BrainVision amplifier reached a significant higher ACC in comparison with the custom-made system during *frowning* and *smiling* (A). There were no significant differences between the healthy and affected sides in VS patients (B). Acceptable ACC was also achieved in high-grade facial nerve palsy (C). *p-values < 0.05.

Significant movement-related EMG activity during *frowning*/*smiling* was detected in 75.2 ± 18.4%/65.3 ± 8.3% by the custom-made EMG device and in 95.9 ± 5.4%/96.4 ± 6.8% by the commercial device in healthy subjects. Classification of movement onset was significantly lower for the custom-made device (*frowning*: *t* = −3.57, *p* = 0.004; *smiling*: *t* = −9.39, *p* < 0.001, Student’s test) (Figure 6A). In postoperative VS patients, there was no significant correlation of the ACC between the healthy and affected side, neither for frontal (*t* = −0.659, *p* = 0.524, Student’s test) nor zygomatic muscles (*t* = −0.756, *p* = 0.465, Student’s test) (Figure 6B). Although there was a trend for a lower movement onset detection rate of the affected side at higher H&B scores, statistical significance was not reached (H = 4.37, *p* = 0.358, Kruskal–Wallis) (Figure 6C). Classification accuracy values of the custom-made device did not differ between healthy subjects and the healthy side of VS patients for *frowning* (*t* = 1.03, *p* = 0.313, Student’s test) and *smiling* (*t* = 1.64, *p* = 0.116, Student’s test).

## Discussion

Despite improved surgical techniques and continuous intraoperative monitoring (Romstöck et al., 2000; Dong et al., 2005; Matthies et al., 2011; Acioly et al., 2013; Sarnthein et al., 2013; Prell et al., 2014; Greve et al., 2020), resection of VS still bears the risk of postoperative facial nerve palsy (Matthies and Samii, 1997; Falcioni et al., 2011), which can significantly impair the quality of life of patients (Ryzenman et al., 2005; Leong and Lesser, 2015). EMG biofeedback is one of the numerous therapeutic approaches for facial nerve rehabilitation in these cases (Booker et al., 1969; Brown et al., 1978; Balliet et al., 1982; Ross et al., 1991; Cronin and Steenerson, 2003; Volk et al., 2015). However, the therapeutic benefit of EMG feedback is not distinct yet, as previous studies showed diverse results (Ross et al., 1991; Nakamura et al., 2003; Dalla-Toffola et al., 2005; Pourmomeny et al., 2014). This may be attributed to the low availability of EMG devices necessitating the biofeedback training to be offered mainly in therapy centers. Low-cost EMG devices could enable standardized home-based training strategies. The present study aimed to evaluate a custom-based EMG device for home-based facial training. Meeting these conditions, we developed a 50 × 115 × 130-mm-sized EMG device that integrates an open-source microcontroller board as well as a sensor chip and costs about $150 in total. Commercial amplifiers, in contrast, cost up to several thousand dollars. The price, size, and simplified handling of the custom-based system allow self-dependent, home-based, and safe biofeedback.

In healthy subjects, there was a significant correlation between EMG amplitudes measured by the custom-made and the commercial EMG device. These data are in good agreement with our hypothesis and prior studies demonstrating a modest to excellent concordance of low-cost to commercial EMG systems by means of, e.g., peak and mean contraction intensity as well as peak duration during isometric and dynamic muscle contractions of large muscle groups (Heywood et al., 2018; Yang et al., 2020). However, while these muscles count a high number of muscle fibers (e.g., approximately 33,000 fibers in the biceps brachii muscle) (Ito et al., 2006), the zygomaticus major muscle consists of only 7,000 muscle fibers. As the EMG amplitude is determined by, e.g., the number of active motor units (Suzuki et al., 2002), it is noteworthy that this is the first study to demonstrate that low-cost PhysComp-based EMG systems can also be used in small muscles. Nevertheless, the detection rate of movement onset showed significantly lower accuracy values of the custom-based EMG device compared with the commercial system. This may be attributed to a higher number of artifacts in the MyoWare compared with the BrainVision data as well as the basic (visual) artifact rejection of our current analysis. Analysis algorithms should be improved to better detect artifacts automatically in future studies (Valderrama et al., 2018; Machado et al., 2021). However, the visual artifact rejection in our study resulted in a comparable exclusion rate of trials for the commercial and custom-based device. This suggests that the lower movement detection rate is not only caused by the artifact rejection technique but also by other amplifier and software characteristics. The lower sampling rate of the custom-based system (i.e., 100 Hz) in comparison with the BrainAmp system (i.e., sampling rate up to 5,000 Hz) should be discussed in this context. According to the Nyquist sampling theorem, sampling rates of 100 Hz enable a reliable detection of frequencies up to 50 Hz, while higher frequencies are affected by aliasing. The frequency spectrum of the facial musculature, on the contrary, ranges between approximately 20 and 500 Hz. Furthermore, previous studies postulated high-pass filtering with a cut off frequency of 15–25 Hz, as lower frequencies seem to be more susceptible to artifacts (e.g., blinking) (Van Boxtel, 2001; De Luca et al., 2010). The low sampling rate of the custom-based amplifier was used to reduce processing and data storage capacity. While for the present time-domain analysis, a low sampling frequency might be acceptable; however, the present sampling rate is not sufficient for other analyses (e.g., in frequency domain). Thus, additional efforts to increase sampling rate are necessary especially as the biofeedback at the same time should necessitate low processing capacity to enable home-based training. The development of different software solutions (e.g., C++ instead of MATLAB) and classification algorithms might improve feedback capabilities in this context, as described by previous EMG studies (Huang et al., 2009; Phinyomark et al., 2013; Singh et al., 2019).

Electromyographic amplitudes recorded in patients after resection of a VS correlated significantly with the degree of the facial nerve palsy according to the H&B score. Hence, surface EMG activity measured by the custom-made device might be an adequate electrophysiological parameter for an objective classification of the grade of the palsy, as previously recommended by Ryu et al. (2018). However, while invasive EMG is a reliable tool to predict unfavorable long-term outcome in facial palsies (Sittel and Stennert, 2001; Grosheva and Guntinas-Lichius, 2007), the predictive power of surface EMG—especially with custom-made systems—still has to be proven.

Classification accuracy of the movement onset did not differ between the healthy and affected side in VS patients and was also admissible in patients with a H&B III–V. As this offers the opportunity of reliable biofeedback, to the opinion of the authors, the presented EMG system might facilitate a large field of applications: (i) training the affected facial nerve (in the early rehabilitation phase), (ii) counteracting facial synkinesias (in the late rehabilitation phase) (Nakamura et al., 2003; Dalla-Toffola et al., 2005; Pourmomeny et al., 2014, 2015), (iii) monitoring rehabilitation success, and (iv) enabling a cost-effective home-based training, which can be performed continuously and over a longer period. This will allow a better understanding of the individual regeneration process and, in case of incomplete recovery, influence decision-making on further surgical interventions like nerve reconstructions. To achieve this objective, therapy schemes and feedback algorithms must be developed, which may be oriented toward the cognitive load theory (Paas and Van Merriënboer, 1994; Schnotz and Kürschner, 2007; Naros and Gharabaghi, 2015; Bauer et al., 2016) and, by determining this, prevent under- and overstraining of the patient and maintain their motivation.

### Limitations of the Study

This proof-of-concept study investigated the validity of a custom-made EMG device using the EMG mean amplitude. While the size and simplified handling of the system enables home-based training, the current device is still too large to be used as a mobile application. A further downsizing by, e.g., using SoCs or smartphones instead of a computer should be aspired in the future. In this context, there are also additional efforts necessary to increase sampling rate while not increasing processing capacity, as the sampling rate of 100 Hz does not capture the whole EMG spectrum. Furthermore, in EMG, not only the mean amplitude predicts muscle function but also several other parameters, e.g., the peak amplitude, the area under the curve (AUC), time to peak or frequency values, such as the peak power, median frequency, or total power, can be analyzed. These parameters allow an improved classification of isometric and static muscle function in terms of, e.g., contractility, speed, and fatigue. They should also be correlated not only with the H&B scale but also with other (regional) facial classification scores (e.g., Sunnybrook Facial Grading Scale and Facial Nerve Grading Scale 2.0). Finally, the applied online feedback algorithm should be improved to allow visualization of facial muscle activity during an active trial instead of feedback after a finished trial. Further studies with a larger patient cohort and longer follow-up period (e.g., 3, 6, 12, and 24 months after surgery) should be carried out.

## Conclusion

The presented EMG biofeedback device is suitable to detect facial muscle activity in healthy subjects and patients with facial palsy after VS surgery and, concurrently, keeps size, cost, and power consumption reasonable. We demonstrated that our device is able to determine the clinical outcome from the EMG acquisition of the user, which is important for both self-dependent, home-based biofeedback and long-term monitoring of the rehabilitation process in an objective manner. Although movement onset detection rates of our device are still lower than in commercial devices, there is potential for improvement by adjusting and integrating sampling rate, filtering, and machine learning techniques. Therefore, software and hardware solutions should be improved, and the clinical impact of a home-based facial EMG biofeedback should be proven in future studies.

## Data Availability Statement

The raw data supporting the conclusions of this article are available and can be provided upon reasonable request.

## Ethics Statement

The studies involving human participants were reviewed and approved by Ethics Committee of the Eberhard Karls University of Tübingen. The patients/participants provided their written informed consent to participate in this study.

## Author Contributions

KM and GN were responsible for the data acquisition, data analyses, statistical analysis as well as the drafting and reviewing of the manuscript. PH and AG provided the measurement devices and were involved in reviewing the manuscript. FG, RS, LT, HH, and MT were involved in the drafting and reviewing of the manuscript. All authors contributed to the article and approved the submitted version.

## Conflict of Interest

The authors declare that the research was conducted in the absence of any commercial or financial relationships that could be construed as a potential conflict of interest.

## Publisher’s Note

All claims expressed in this article are solely those of the authors and do not necessarily represent those of their affiliated organizations, or those of the publisher, the editors and the reviewers. Any product that may be evaluated in this article, or claim that may be made by its manufacturer, is not guaranteed or endorsed by the publisher.

## Abbreviations

ADC: analog-to-digital converter
CMRR: common-mode rejection rate
EMG: electromyography
H&B: House and Brackmann Scale
LI: laterality index
MEP: motor evoked potential
PhysComp: physical computing platform
SUNNY: Sunnybrook scale
SoC: system-on-a-chip.
